# An integrated study to decipher immunosuppressive cellular communication in the PDAC environment

**DOI:** 10.1038/s41540-023-00320-6

**Published:** 2023-11-10

**Authors:** Gülben AVŞAR, Pınar PİR

**Affiliations:** 1 Department of Bioengineering, Gebze Technical University, Kocaeli, Turkey; 2 Turkish Academy of Sciences, Ankara, Turkey

**Keywords:** Cancer, Computational biology and bioinformatics, Immunology

## Abstract

Pancreatic ductal adenocarcinoma (PDAC) is one the most aggressive cancers and characterized by a highly rigid and immunosuppressive tumor microenvironment (TME). The extensive cellular interactions are known to play key roles in the immune evasion, chemoresistance, and poor prognosis. Here, we used the spatial transcriptomics, scRNA-seq, and bulk RNA-seq datasets to enhance the insights obtained from each to decipher the cellular communication in the TME. The complex crosstalk in PDAC samples was revealed by the single-cell and spatial transcriptomics profiles of the samples. We show that tumor-associated macrophages (TAMs) are the central cell types in the regulation of microenvironment in PDAC. They colocalize with the cancer cells and tumor-suppressor immune cells and take roles to provide an immunosuppressive environment. LGALS9 gene which is upregulated in PDAC tumor samples in comparison to healthy samples was also found to be upregulated in TAMs compared to tumor-suppressor immune cells in cancer samples. Additionally, LGALS9 was found to be the primary component in the crosstalk between TAMs and the other cells. The widespread expression of P4HB gene and its interaction with LGALS9 was also notable. Our findings point to a profound role of TAMs via LGALS9 and its interaction with P4HB that should be considered for further elucidation as target in the combinatory immunotherapies for PDAC.

## Introduction

Pancreatic ductal adenocarcinoma with its <10% 5-year survival rate is one the most devastating cancer types. The poor prognosis is mainly associated with the lack of reliable diagnostic and prognostic biomarkers, low surgical resections rate, rigid stroma structure and high immunosuppressive infiltrate^1–4^. These factors play significant roles in promoting resistance to therapies, making it challenging to overcome immune checkpoint blockade and penetrate the solid stroma^5–7^. The clinical trials of the immune checkpoint inhibitors as programmed cell death protein 1 (PD-1) and cytotoxic T lymphocyte-associated protein 4 (CTLA4) have promising results with other cancer types, whereas they have been unsuccessful in PDAC^6,8^. Hence, the existing therapy strategies cannot provide complete treatment of PDAC, more effective approaches are urgently needed. Recently, the need to understand the tumor microenvironment (TME) and immunosuppressive infiltration have gained more and more importance to reveal the new agents and therapies for the treatment of PDAC and increased survival rates.

The transcriptomics technologies have been widely used to discover and understand the cellular compositions in the microenvironment, cellular response to the perturbations, and cellular states of each cell^9,10^. Integrated analysis of the transcriptomics datasets which have been produced by different strategies such as bulk RNA-seq, scRNA-seq (SC) and spatially resolved transcriptomics (spatial transcriptomics, ST) may lead to more systematic and holistic knowledge discovery by overcoming the limitations of each. Several challenges such as the missing value problem in SC and ST datasets, the lack of cell type detection with bulk RNA-seq and ST technologies, the lack of locational information in bulk RNA-seq and SC datasets hinder their potential to reveal the profile of the samples. Additionally, the stress which is generated on the cells during the experimental steps may cause variations in the transcriptional profiles. Therefore, the analysis for the individual cell, the effect of locational properties of cells, and the impact of the cell in the bulk can be studied by using scRNA-seq, spatial transcriptomics and bulk RNA-seq integratively^2,10,11^.

LGALS9 which encodes galectin-9 (Gal-9) is a member of galectin family of animal lectins and is found in cytosol, nucleus, plasma membrane and extracellular regions^12^. It is suggested to be an immune checkpoint which binds to PDCD1 (PD-1), HAVCR2 (TIM-3), CLEC7A (dectin-1) and CD44 that regulates the immunological response by driving T cells to apoptosis and tolerogenic macrophage programming^13–16^. The overexpression of LGALS9 is associated with the tumor development, metastasis and poor prognosis^17^. It is also proposed as a marker, reported to be upregulated in PDAC samples and to be associated with the poor prognosis^3,18^. A recent study suggested that the co-inhibition of LGALS9 and CD274 (PDL-1) resulted with a more effective tumor growth inhibition in PDAC^19^. The disruption of LAGLS9-CLEC7A axis was reported to enhance the survival rate and decreased the tumor growth. However, the neutralization of LGALS9 alone failed to suppress the tumor in PDAC^15^. Therefore, the interactions of LGALS9 in the TME has gained much attention to elucidate efficient immunotherapy strategies. LGALS9 also binds P4HB (PDIA1) which results in cell migration and T cell inhibition^20–23^. P4HB is a protein disulfide isomerase and a chaperone that is found in cytosol, mitochondria, extracellular space, and on the cell surface^21^. P4HB was found to be overexpressed in various cancer types including bladder cancer, renal clear cell carcinoma, hepatocellular carcinoma, and glioblastoma^24–27^. Although, several transcriptome studies showed that P4HB is downregulated in PDAC, only one study reported the upregulation of P4HB for PDAC^28–31^. On the other hand, the expression of this gene was indicated to be related with the stage of the cancer; in the first and fourth stages it is downregulated while upregulation is reported in the second and third stages of PDAC^32^. The cell surface P4HB is associated with the adhesion and migration of T cells, cancer cells and endothelial cells, HIV infectivity, and chemoresistance^22,23,33^.

In the present study, we aimed to discover the cellular interactions in PDAC using an integrative approach for the different transcriptional states that are potentially associated with immunosuppressive TME. Cellular heterogeneity in PDAC was discovered by using paired ST and SC datasets which were derived from the same tissue of the same patients. A colligation strategy was taken on to discover the cellular heterogeneity and cell-to-cell interactions in the TME (Supplementary Fig. 1). The SC and ST datasets were processed separately, the communication results were analyzed by assessing the number of interactions and most frequent pairs which was constructed between and within the cell types in SC and domains in ST. The most frequent partner which was used in communication pairs was also discovered and further analyzed to reveal the interplay of cells in PDAC. Cellular communication analysis revealed the tumor-promoting immune cells, especially TAMs, as the key cell type to generate an immunosuppressive TME with cancer cells. LGALS9 was detected as the most used constituent in the connections between the cells after excluding the putative ECM-bound interactions. Additionally, the LGALS9 interaction with P4HB was widely detected which is mostly associated with the cell migration. Although the downregulation of P4HB in bulk datasets was observed, its upregulation and high abundance on throughout the tissue samples were shown by ST and SC datasets for PDAC. The outcomes suggested the need for further analysis for the combinatory therapies which will target LGALS9 and its interaction with P4HB to provide increase in the efficacy in PDAC treatments.

## Results

### Cellular composition in PDAC

**Fig. 1:**
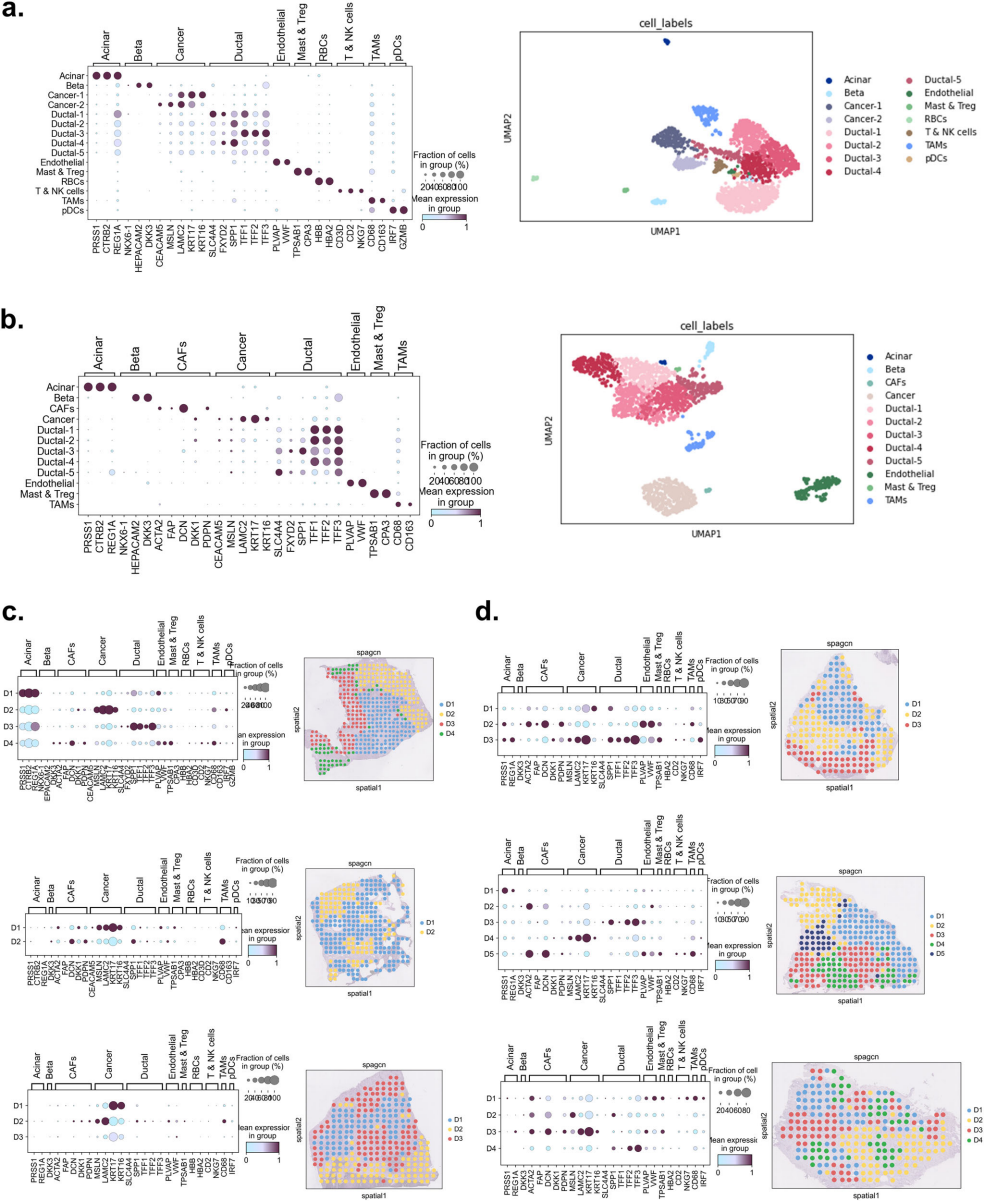
Cellular heterogeneity in PDAC tissues. The cell clusters and cell type specific marker genes in **a** scA and **b** scB. The domains and cell type specific genes in **c** stA and **d** stB.

The cell types were identified using SC datasets by inquiring the cell type specific marker genes (Fig. 1a, b). In scA and scB, we detected 5 ductal cell subclusters in both datasets. There were two cancer clusters in scA, while only one cancer cluster in scB. Additionally, we could not detect the clusters for T cells and NK-cells, RBCs, and pDCs in scB. On the other hand, gene markers for fibroblast were obvious in only scB. Besides the T cells and NK-cells, the clusters which consist of TAMs, mast cells and Tregs were also detected as the immunological cell types. While T cells and NK cells were indicated as the tumor-suppressor immune cells, the other immune cell types were defined as the tumor-promoting immune cells^34,35^. The defined cell types were also validated with an independent study (named as scC in this study) in which data was collected from 14 PDAC patients (Supplementary Fig. 2).

To characterize the ductal cell clusters, functional enrichment analysis was performed on the differentially expressed genes of each cluster (Supplementary Fig. 3). The analysis resulted in the cell survival against the apoptosis and regulation of cellular communication processes for Ductal-1. The Ductal-2 cells were defined with the GO terms of developmental, tube morphogenesis and cell motility processes. The GO terms related to cellular respiration and adhesion, the epithelial cell differentiation, and cytokine-mediated signaling pathway were associated to Ductal-3 cells. The immune responses, homeostatic process, and lipid metabolic process were enriched in the cluster of Ductal-4 cells. And finally, high mitotic cell activities accompanying the transcript regulation were detected for Ductal-5 cell cluster. Further, the subpopulations for the ductal cells were identified: CRISP3 and CFTR expressing centroacinar ductal cells^36^; TFF1, TFF2 and TFF3 expressing terminal ductal cells; APOL1 expressing mobile ductal cells; SPP1, SERPING1 and MHC class II genes (CD74, HLA-DRB1 and HLA-DRB5) expressing antigen-presenting ductal cells^37^; and TUBA1B and HMGB1 expressing proliferative ductal cells. In scA and scB, Ductal-1, Ductal-2, Ductal-3, Ductal-4 and Ductal-5 cell clusters were defined as the centroacinar, mobile, terminal, antigen-presenting, and proliferative ductal cells, respectively.

### Cellular distribution in spatial design

The domains which are defined as the genetically and histologically coherent regions in the tissue were identified using ST datasets. By integrating the gene expression profiles and the histological image pixel intensities of each spot, SpaGCN determined the domains on the tissue sample. ST and SC datasets were expected to have similar cell types as they were obtained from the same tissue of the same patients. Therefore, the marker genes of each cell type which were detected in SC datasets were plotted to detect the cell types in the domains (Fig. 1c, d). Due to the lack of expression of cell type specific genes, the clusters for CAFs and T & NK cells were not detected in the scA and scB, respectively. On the other hand, the cell-type specific marker genes of these cell types were observed in ST datasets. PDAC tissues are characterized by having a dense stroma which is called desmoplasia with a substantial fibroblast accumulation^1^. Even though CAFs were not found in the SC dataset of patient A (scA), they were detected in ST datasets both in stA and stB samples. As expected, fibroblasts, being one of the main constituents of the stroma^38^, were located almost everywhere in the tissues. Also, the marker genes of CAFs were high in the domains where the cancer-specific marker genes are overexpressed (see stA2, stA3, stB1, and stB3 in Fig. 1c, d). Compared to SC, the ST may be more effective in identifying the cell types that are truly present in the tissue, because RNA is extracted from the cells which have still been connected to both the ECM and to one another. Additionally, since there is no tissue dissociation step, the spatial context of the cells is preserved. However, it is not possible to label the spots with a specific cell type due to the possibility of co-localization of several cell types in the spots. Indeed, the marker genes of several cell types (i.e., CAFs, ductal cells, cancer cells, and TAMs) were observed in the spots of all domains, as expected. To study the co-localization of specific cell types, each domain was analyzed for the abundance of cell type specific marker genes in the same domain in each dataset. Because each domain has its own characteristics in all datasets, the domains with the same label do not refer to the same profile in different datasets (i.e., D1 does not represent the same biological profile in different datasets). The detection of domains was carried out with dataset-based approach which generated unmatched domains with same labels in different datasets. Therefore, the analysis of domains was performed independently by ignoring the domain label.

Ligand-receptor (L-R) pairs are the main components of the cellular crosstalk complex. The pairs in the datasets were detected by using CellPhoneDB based on their gene expression profiles. The domains in which the cancer cell marker genes are highest expressed were detected. The ligand and receptors in self-interaction in each domain were subjected to functional enrichment analysis. It was found that the domains in which the cancer marker genes are highly expressed are involved mainly in vascularization, cell migration, and ECM reorganization (Supplementary Fig. 4). If the T & NK cell marker genes are also abundant besides the cancer marker genes in a domain, T-cell activation and negative regulation of cytokine production have also been found as enriched GO terms. Similarly, cell motility, blood vessel formation, and ECM reorganization to confer tensile strength processes were included in the interactions of domains with highest fibroblast marker genes and cancer marker genes. The ECM organization GO term contains genes taking part both in the degradation and in the strengthening of ECM. The degradation is required to form blood vessels and provide permissibility for cellular migration. Additionally, strengthening of ECM to maintain the tensile strength is important for a dense stroma^1^. The observed processes were supported by the literature of the known PDAC characteristics such as immunosuppression, ECM remodeling, angiogenesis, and desmoplasia^1,2,38,39^.

### Cellular interaction profiles

**Fig. 2:**
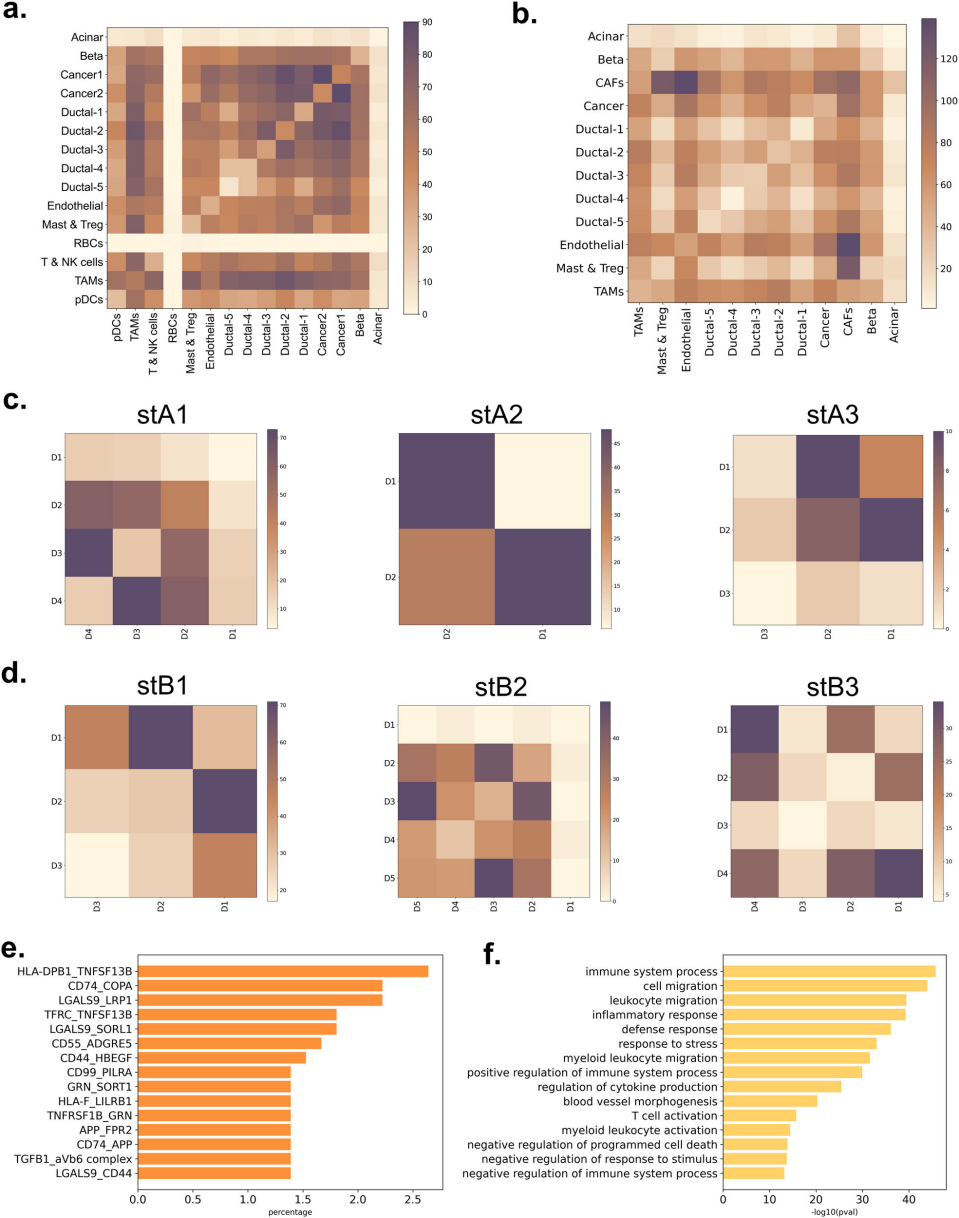
Communication profiles in the datasets. The number of interactions between and within the cell types in **a** scA and **b** scB, and the domains in **c** stA and **d** stB (The color intensity in the heatmaps shows the number of observed pairs in the corresponding dataset). **e** The most frequent 15 L-R pairs (the x-axis refers to the percentage of the corresponding L-R pair in the complete list of interactions), **f** The enriched GO terms for the proteins with interactions between ductal cells and TAMs.

scRNA-seq technologies enables profiling of the tissue samples at single cell resolution. Hence, inference of cell-cell communication is possible besides revealing the cellular heterogeneity, detection of rare cells and elucidation of gene regulatory networks in individual cells. The analysis of the expression values of ligand and/or receptor coding genes facilitates the discovery of constructed L-R pairs between and within the cell types^40,41^. L-R analysis with scRNA-seq datasets using CellPhoneDB provided the interpretation of cell-to-cell communication in PDAC. In addition to ductal cells, the interactions with cancer cells, CAFs, endothelial and TAMs were observed in considerable numbers in all SC datasets (Fig. 2a, b and Supplementary Fig. 2b). In scA and scC, cancer cells were found to mostly interact with other cancer cells and ductal cells, as the highest number of interactions were obtained between these cell types (Fig. 2a and Supplementary Fig. 2b). And also, cancer cells were observed to have a large number of interactions with TAMs and endothelial cells, in scA. In scB, the cancer cells were found to mostly interact with CAFs, endothelial cells and TAMs based on number of interactions, respectively (Fig. 2b). Both ductal cells and acinar cells have potential to develop PDAC^42^. Because the number of interactions of acinar cells are lower compared to others, we focused on ductal cells to reveal the interactions that may be important for PDAC progression. The number of interactions showed that ductal cells were found to be mostly interact with TAMs and cancer cells in scA, respectively (Supplementary Fig. 5). In scB sample, CAFs, endothelial cells and TAMs mostly interacted with ductal cells, respectively. TAMs were found as one of the top interactors of ductal cells in both datasets which may point out the roles of TAMs in cancer progression and control^43^.

Similar to scRNA-seq, the communication profiles were also revealed for the domains in ST datasets by using CellPhoneDB (Fig. 2c, d). If the acinar cell specific marker genes are high in a domain, the number of interactions were observed to be the lowest compared to other domains in the same dataset. When we focused on the domains with larger number of interactions, it was seen that if there was a clear ductal cell domination (see stA1, stB2 and stB3 in Fig. 1c, d), these domains were one of the partners of these interactions (D3-D4, D3-D5, and D1-D4 in stA1, stB2, and stB3, respectively). The other partner domain for these interactions were observed to contain genes of CAFs, TAMs and endothelial cells in the majority of the cases. An addition to outcomes for ductal cells in ST datasets, they were also found to be interacting with TAMs in both scA and scB, as indicated above. The number of interactions between ductal cells and TAMs were found to be significantly higher than the average total number of interactions in all datasets (Supplementary Fig. 5). This may indicate that the role of ductal cells in the immune infiltration is significant. Thus, the 15 cell-cell interactions with highest scores (top 15 interactions) between the ductal cells and TAMs were investigated further (Fig. 2e). These interactions were found to be associated with the processes of cell adhesion and immune regulation. However, contradictory terms were obtained for the immune system activation which may refer to the effort of ductal cells to activate immune response while tumor-promoting immune cells work to negatively regulate the immune system (Fig. 2f).

It is worth to note that the possible presence of multiple cell types in each spot may give rise to a lower number of interactions compared to SC datasets, because each spot gives only one gene expression profile (analogous to data from a mini bulk sample) in which we may not be able to detect the interactions within spots. The transcriptomics are collected from the cells which have still been attached to both the ECM and other cells in ST datasets. Therefore, the collagen and integrin involved interactions were expected to be overrepresented in these datasets. Not surprisingly, the interactions between the collagen proteins (i.e., COL1A1, COL1A2, COL3A1, and COL4A1) and integrin complexes (i.e., a1b1, a2b1, a3b1 and a4b1) were the most common interactions in the ST datasets.

**Fig. 3:**
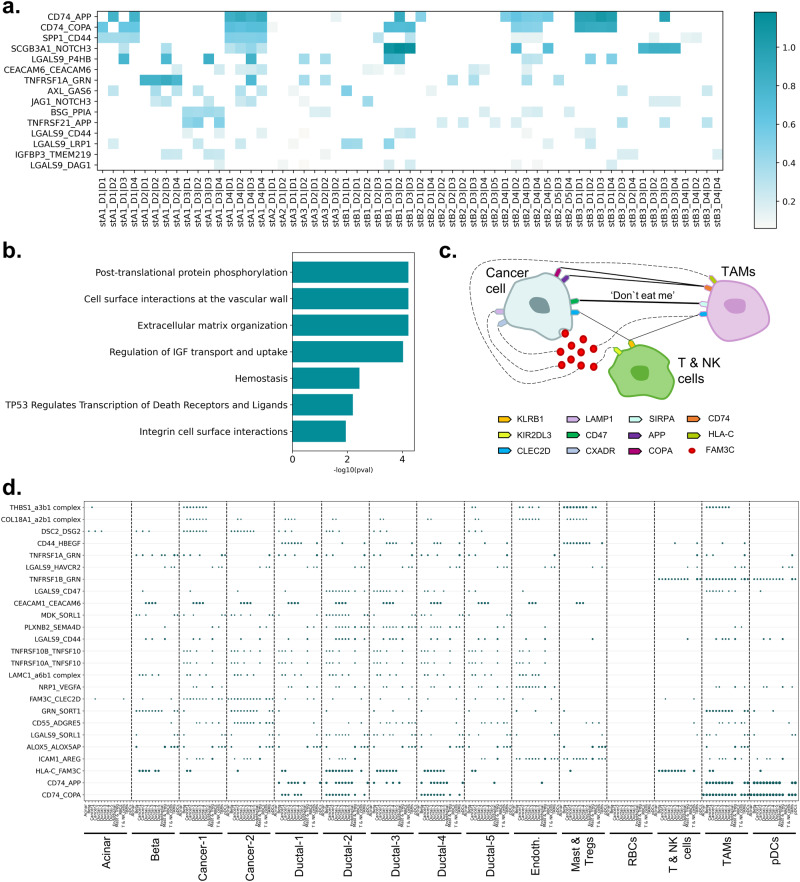
The observed cellular interactions. **a** The top 15 L-R pairs in ST datasets (the color intensity shows the score of the interaction in the corresponding dataset), **b** the associated processes of top 15 L-R pair elements in ST datasets, **c** the communication between cancer cells, tumor-promoting immune cells and tumor-suppressor immune cells, **d** the top 25 L-R pairs in scA.

The most frequent 15 L-R pairs were detected after excluding the integrin-involved interactions in ST datasets to eliminate the interactions which cannot be classified to be cell-to-ECM or cell-to-cell (Fig. 3a). The elements of the pairs were found to be associated with the cellular reactions of protein and ECM organization, homeostasis, attachment to the blood vessel for the nutrition, cell viability, and cellular growth (Fig. 3b) The pairs between CD74 - APP, CD74 - COPA, SPP1 - CD44, SCGB3A1 - NOTCH3, and LGALS9 - P4HB were the top 5 in the mostly observed L-R pairs within and between the domains. These pairs take part in angiogenesis and immune regulation in the tumor microenvironment^44^ via their association with immune checkpoint inhibitors^45^. APP and COPA are suggested to play promoting roles in tumor progression and metastasis^39,46,47^. The binding of the ligands to CD74 on immune cells is associated with the immunosuppressive context^48,49^. SPP1 was shown to have critical roles in cancer progression, metastasis and therapy resistance, and was suggested to be a T-cell activation inhibitor^50–53^. CD44 is a cellular adhesion molecule and highly associated with the cancer stem cell population and mesenchymal phenotype^49,52,54^. In PDAC, the cancer stem cell population of the tumors was found to be induced by CAFs via SPP1-CD44 interaction. The overexpression of SPP1 and CD44 is associated with poor prognosis in PDAC patients^52^. Besides the regulation of epithelial cell proliferation and differentiation, SCGB3A1 has a role in cellular membrane organization and in the local immune response in the lung^55^. NOTCH3 is formerly implicated in angiogenesis, and recent findings indicated its roles in tumorigenesis, tumor maintenance and resistance to therapies. The expression of NOTCH3 is increased in hypoxic conditions and associated with poor prognosis in several cancers^56,57^. In PDAC, NOTCH3 is overexpressed and strongly associated with the vascular invasion, metastasis, and resistance to chemotherapy^57^. P4HB is associated with the adhesion and migration of cancer cells and platelets by the PDI reduction of disulfide bonds in β-integrins^20,58^. The interaction of LGALS9 and P4HB was shown to increase T cell migration and viral entry^20^. Hence, it may be concluded with that cellular communication in PDAC tissue is highly associated with the cancer survival via immunosuppression and cellular reorganization to accompany hypoxia and desmoplasia. The expression patterns of the related genes were expressed in a large range of levels throughout the ST domains (Supplementary Fig. 7).

Our results (Fig. 3d, Supplementary Fig. 2d, and Supplementary Fig. 6) suggest that TAMs were suppressed via activation of the CD74 axis which also induced the expression of hypoxia-related genes to promote cell survival. The localization of the tumor-promoting immune cells might be supported with the interaction of SPP1 and CD44 in the tumorous ECM. SPP1-induced CD44 cleavage results in the expression of hypoxia-related gene^49^, therefore maintenance of the cellular vitality might be provided by SPP1 and CD44 interaction in the hypoxic environment of the PDAC tissue.

SCGB3A1 is a surfactant protein which reduces the cell surface tension, so the collective cell movement is induced from aggregate surface region to aggregate core region^59^. The interaction we find between the SCGB3A1 and NOTCH3 might provide the inward vascular invasion into the rigid tumorous tissue in PDAC. Treg differentiation and inhibition of T cells might be done by the secretion of LGALS9 into the microenvironment^13^. Also, the interaction of LGALS9 with P4HB might contribute to cellular motility and the angiogenesis^21–23^.

The immunoregulation identified by ST datasets were also supported by the SC datasets. The most common 25 L-R pairs showed that the top 3 pairs were CD74-COPA, CD74-APP, and HLA-C-FAM3C in both scA and scB (Fig. 3d, and Supplementary Fig. 6). Interestingly, cancer cells do not prefer to construct interactions as a sender cell type with other cells using these pairs. On the contrary, these pairs were highly observed in the interactions of cancer cells as a receiver cell type. As an addition to CD74, COPA, and APP which were mentioned above, FAM3C which was observed to be expressed by cancer cells in the HLA-C-FAM3C interaction is identified as a specific gene for the epithelial-to-mesenchymal transition (EMT) and correlated with poor prognosis^60,61^. It is highly expressed in malignant cells including in PDAC^5^. We found that FAM3C interacted with several other receptors such as PDCD1, CLEC2D, FFAR2, CXADR, LAMP1, and KIR2DL3 which are associated with immune response, tissue homeostasis, organ development and lysosome^62–64^. In addition, the interactions of KIR2DL3 and FAM3C, and KLRB1 and CLEC2D were detected between cancer cells and T & NK cells (Fig. 3c). KIR2DL3 and KLBR1 are known as the receptors which inhibit the NK cell-mediated killing^65^.

In almost all datasets, the interaction of cancer cell types with the TAMs clusters has a high number of significant L-R pairs which indicates the high immunological activity in the tissue (Fig. 3d, Supplementary Fig. 2c, and Supplementary Fig. 6). Because the mentioned three L-R pairs (CD74-COPA, CD74-APP and HLA-C-FAM3C) were observed extensively in the interactions from TAMs to cancer cells, it can be suggested that CD74 and HLA-C are highly active in TAMs.

Additionally, the CD47-SIRPA interaction is known as the ‘don’t eat me’ signal which healthy cells send to macrophages to avoid from phagocytosis^66,67^. CD47-SIRPA pair was observed between cancer cells and TAMs in our analysis. Thus, cancer cells manipulated the environment by regulating the phagocytosis for the evasion of immune surveillance. These results suggested that the mentioned interactions were highly effective to escape from the immune system by inhibiting the T cells and NK cells in PDAC.

For their ability of traveling more efficiently in TME, presenting the antigens and generating endogenous responses, targeting of myeloid cells were suggested for the adoptive cell therapies by several studies^68–70^. Similarly, the cell type distribution profiles (Fig. 1) and the cellular communication profiles (Fig. 2) showed that the tumor-promoting immune cells, especially TAMs, have a critical role in the tumor ECM. We find that TAMs are colocalized with T & NK cells and cancerous cells which may indicate the inhibition of tumor-suppressor immune cells and tumor promoting activity, respectively. These outcomes suggested that the macrophage targeted combinatorial strategies may provide more effective results for the treatment of PDAC patients.

**Fig. 4:**
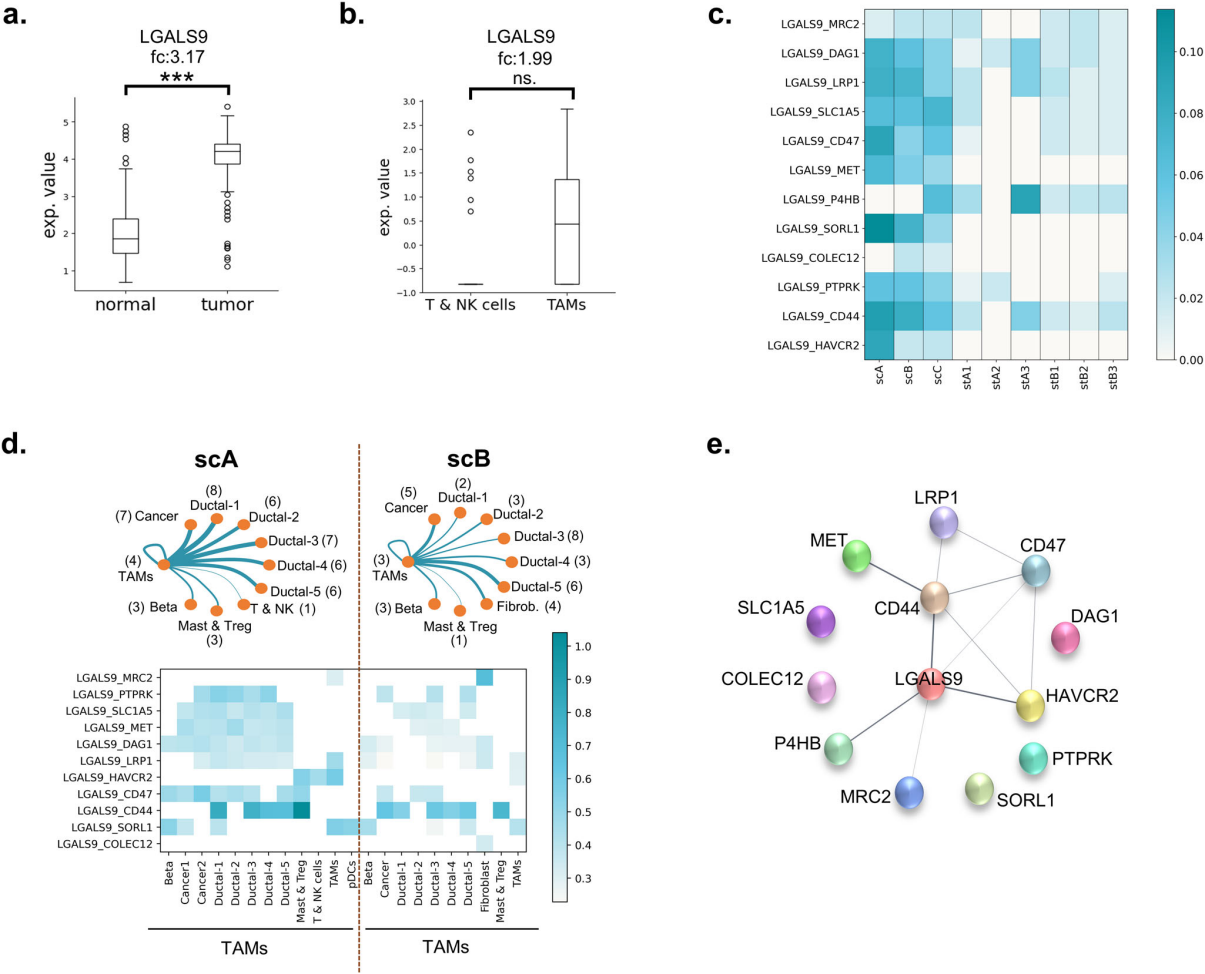
LGALS9 in TME. **a** LAGLS9 overexpression in TCGA-PAAD compared to GTEX datasets. **b** LGALS9 overexpression in PDAC patients with the comparison of TAMs and T & NK cells. **c** the LGALS9-participated interactions in SC and ST datasets. **d** The distribution of LGALS9 interactions within TAMs and the other cell types (the number between the parentheses indicates the number of interactions with TAMs). **e** PPI network of LGALS9 and its receptors which were found in SC and ST datasets. (The color intensity in the heatmaps shows the score of the pair in the corresponding dataset).

When we excluded the integrin-included components to eliminate the probability of interaction to be constructed with ECM, LGALS9 was found to be the ligand that is mostly used in the interacted pairs in ST datasets. Likewise, it was one of the most preferred ligands in SC datasets. We showed the upregulation of LGALS9 in PDAC patients using bulk RNA-seq datasets (TCGA and GTEx datasets) (Fig. 4a) as reported previously by the several studies^3,18^. Also, LGALS9 was upregulated in TAMs compared to T & NK cells in both scA and scC datasets (Fig. 4b and Supplementary Fig. 2d). Twelve LGALS9-included pairs were found, four of them were observed only in SC datasets (Fig. 4c). These interactions have been suggested to be associated with the ECM remodeling, cellular adhesion, cell migration, and cellular uptake of LGALS9^20,71–77^. Additionally, LGALS9-HAVCR2 interaction which is associated with the T-cell inhibition^78^ was one of the observed 12 pairs. The analysis of cellular communication with SC datasets showed that the L-R pairs of LGALS9 are highly abundant in the communication of the TAMs with mostly cancer cells, ductal cells, and TAMs themselves (Fig. 4d). Thus, we hypothesized that TAMs may act as a main regulator in TME with the cell-cell interactions via LGALS9.

**Fig. 5:**
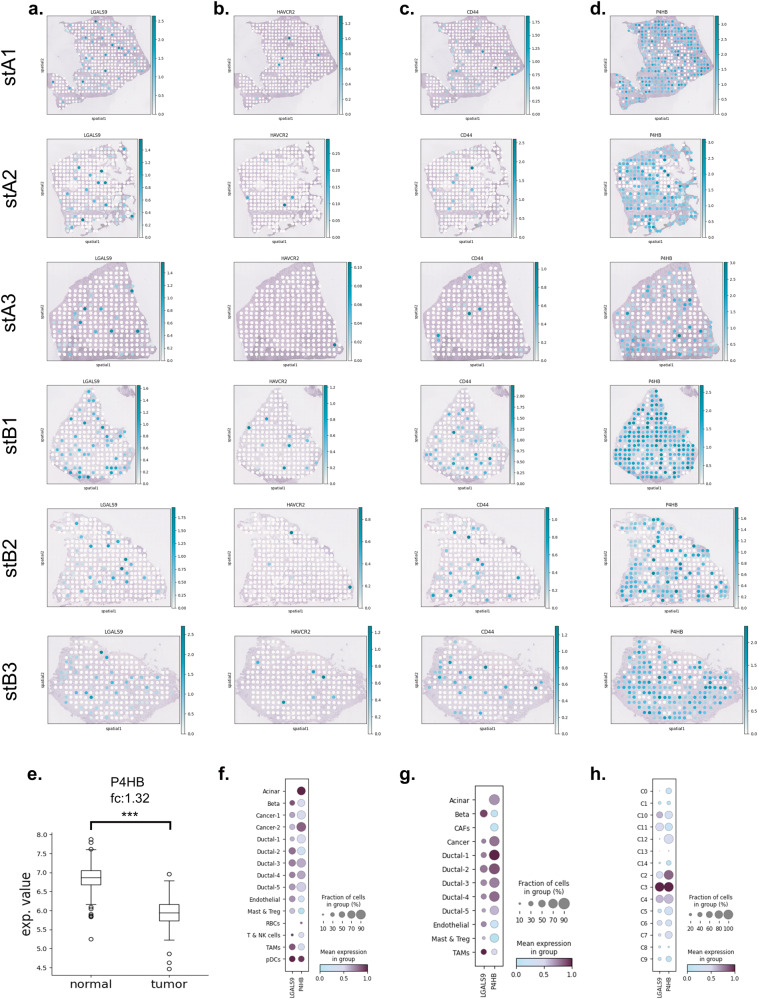
Spatial distribution of the genes. The expression profiles of **a** LGALS9, **b** HAVCR2, **c** CD44, and **d** P4HB gene on tissue samples (The color intensity refers to the expression value of the corresponding gene in the dataset.). **e** The regulation of P4HB in bulk datasets. The expression profiles of LGALS9 and P4HB in **f** scA, **g** scB, and **h** scC.

The 12 LGALS9-binding proteins were incorporated into one PPI network in which the color intensity of edges associated with the confidence score (Fig. 4e). Within the 12 interactions, only the interactions of LGALS9 with P4HB, CD44 and HAVCR2 have a confidence score that is higher than 0.9. The spatial distribution of the 4 genes have been visualized on the H&E-stained images which the mRNA molecules have been collected for ST datasets (Fig. 5a–d). While LGALS9, HAVCR2 and CD44 have been observed in a few spots and at the low level of expression counts, P4HB was found to be widely expressed in most regions of the tissue samples. As mentioned before, the LGALS9-P4HB was the most frequent interaction among interactions of LGALS9 in ST datasets (Fig. 3a). This interaction was reported to increase the cellular migration by regulation of cell membrane redox status of T cells^20^. Similarly, the cell surface P4HB was associated with the migration of cancer cells and endothelial cells and also the chemoresistance^21–23^. Interaction with LGALS9 might lead P4HB to exchange the exofacial properties by regulation of the membrane redox status to provide cellular migration.

**Fig. 6:**
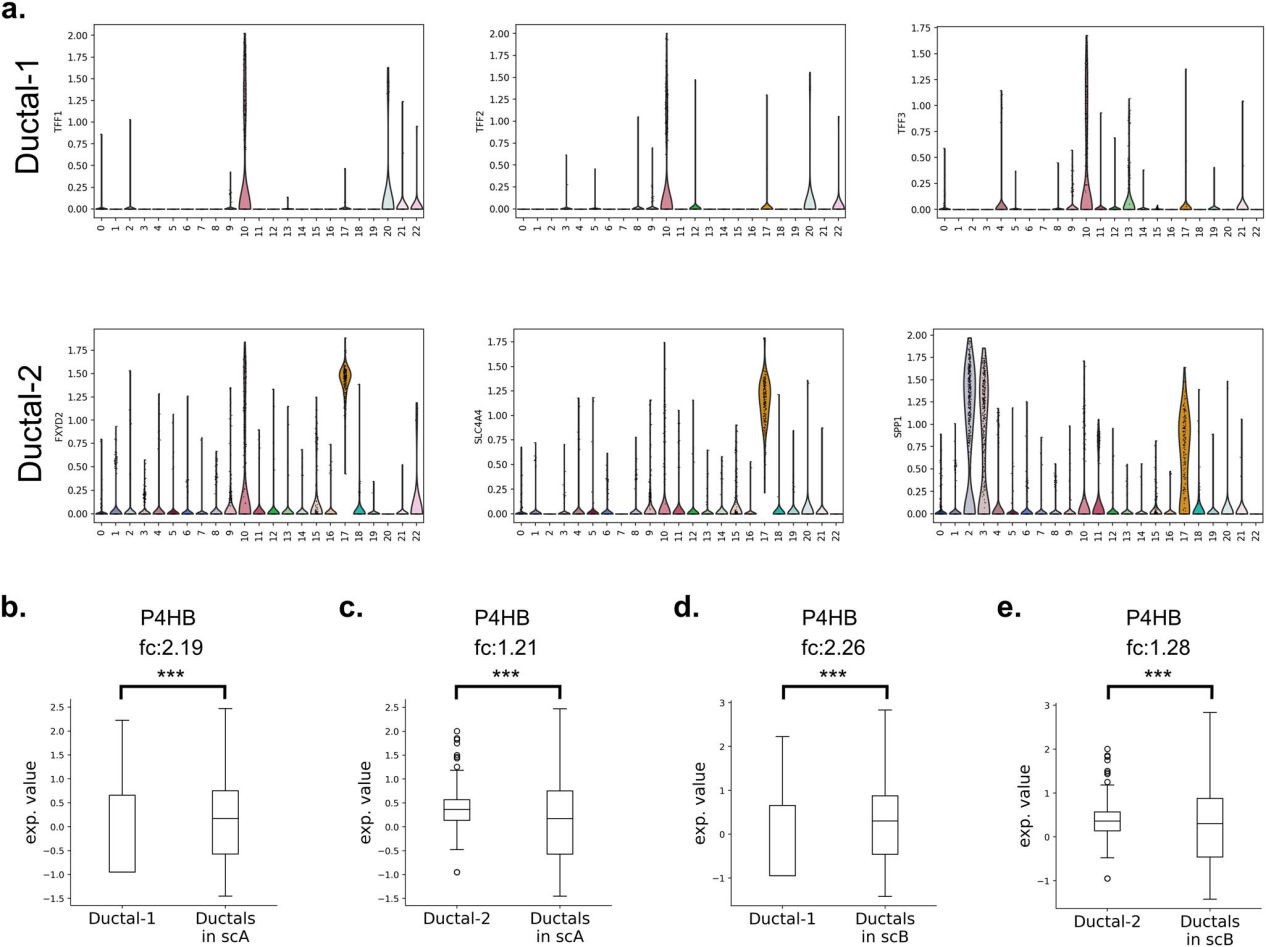
P4HB profile in SC datasets. **a** The ductal cell clusters in adj dataset. The regulation of P4HB in comparison of **b**, **c** adj with scA, and **d**, **e** adj with scB.

P4HB was reported to be downregulated in PDAC using microarray and TCGA datasets^28–30^. Here, we also showed its decrease in tumor samples via comparison of TCGA-PAAD and GTEx bulk RNAseq datasets (Fig. 5e). On the other hand, the upregulation of P4HB has been reported by a few studies at the proteomics level in pancreatic cancer and pancreatic islets of type I diabetes patients^79^. While the expression level of LGALS9 was high mostly in TAMs, and also cancer cells and ductal cells, P4HB was found to be widely expressed by cancer cells, ductal cells and TAMs, respectively in our SC datasets (Fig. 5f–h). To reveal the regulation of P4HB in cell type level, we compared PDAC tumors (scC) with its adjacent normal samples (adj). In the adj dataset, two ductal clusters were identified. One cluster expresses TFF1-3 genes at a high level, while the other cluster expresses other ductal-specific genes (Fig. 6a). We concatenated all ductal cell clusters into one cluster in scA and scB, the differential expression was elucidated for P4HB in these clusters. In the tumor samples, the expression of P4HB was significantly higher than the ductal clusters of adjacent normal samples (Fig. 6b–e). As mentioned before, the different technologies can be integrated to eliminate the limitations of each other. Here, we showed that although downregulation of P4HB was observed via bulk RNA-seq datasets, its upregulation in SC datasets and ubiquitous expression pattern of the gene throughout the tissue sample with ST datasets is evident. When expression profile of LGALS9 and P4HB and their highly active interaction are taken into consideration, it can be concluded that they have crucial roles in PDAC. These findings suggest that a multi-target strategy which targets both the immune checkpoint LGALS9 and protein disulfide isomerase P4HB may offer more effective immunotherapy in PDAC treatment.

## Discussion

In this study, we examined the cellular communication in the PDAC tumor microenvironment to reveal the underlying mechanism of cellular interactions. Reanalysis of the spatial transcriptomics and single cell RNA sequencing datasets which were retrieved from the same tissue of same patients, showed that the combinative analysis of transcriptomics generated by different technologies can address the challenges of each technology. scRNA-seq offers single-cell resolution profiles, however it lacks the locational information, and some cell types cannot be identified due to the missing value problem in marker genes. Spatial transcriptomics provide gene expression profiles with the locational details, but because the size of spots on which the RNAs are captured is larger than the size of a single cell, each profile comes from multiple cells. As an addition, both scRNA-seq and ST technologies produce sparse matrices. The integration of these two technologies can help to eliminate the limitations of each other. Here, we deciphered the cellular heterogeneity landscape in PDAC tumor samples that the SC and ST datasets were derived in parallel. The domains on the tissue samples were revealed using ST datasets. Additionally, the communication profiles between and within the domains in ST datasets were revealed, and the detailed investigation which is based on the cell types was supported with SC dataset.

**Fig. 7:**
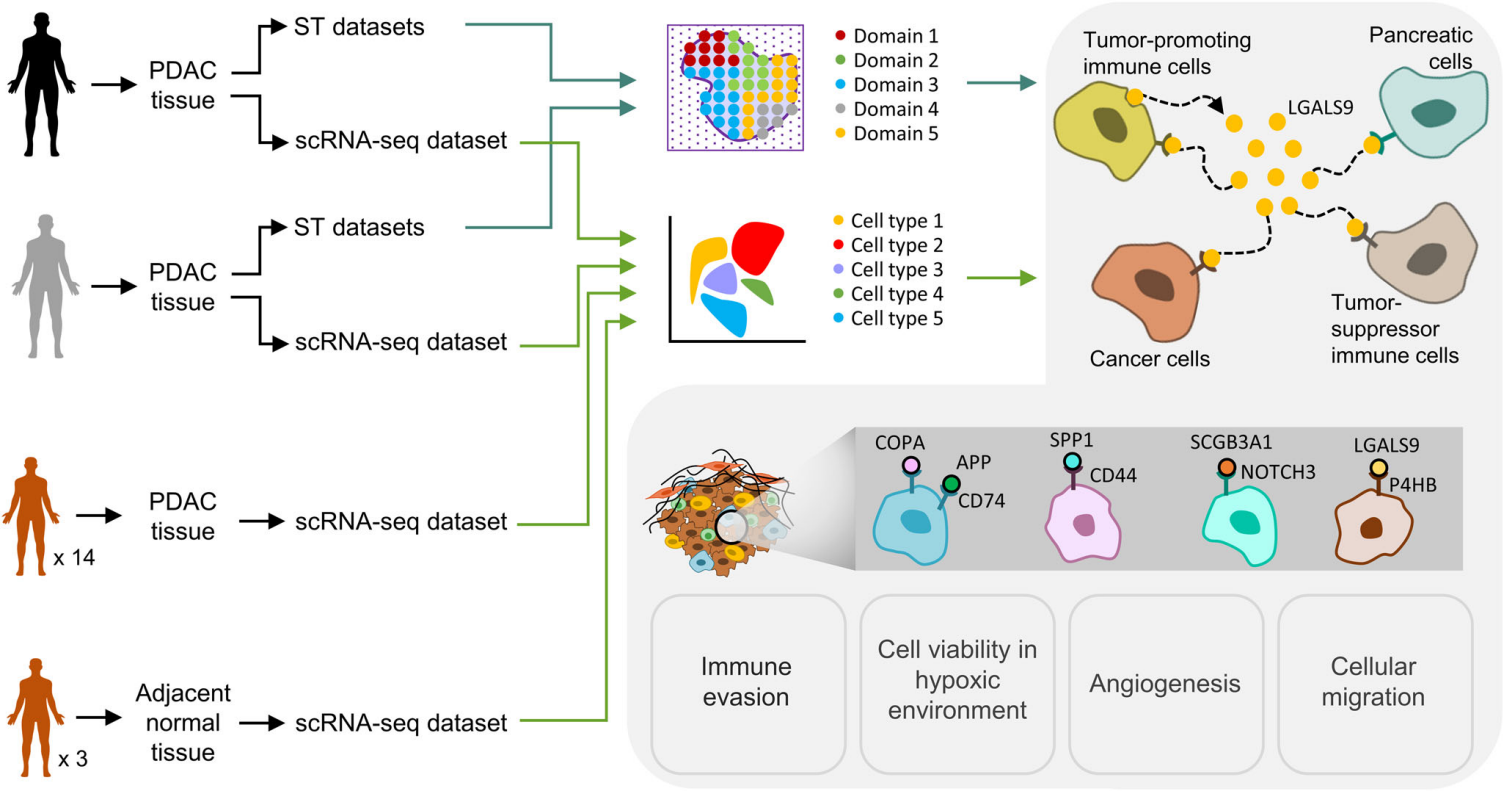
The overview of the study. The observed landscape for the immunosuppressive PDAC TME.

The CAFs, cancer cells, and TAMs were found to be spread throughout the tissue samples. The interactions of cells with the ECM were the mostly observed interaction type, as expected. Besides the ECM-bound interactions, the pairs which were associated with the inhibition of tumor-suppressor immune cells, expression of hypoxia-related genes, angiogenesis, and vascular permeability to sustain cellular viability were frequently observed (Fig. 7). The cancer cells manipulate TAMs by sending ‘don’t eat me’ messages and cooperate with TAMs to suppress the T cells and NK cells. Additionally, TAMs were identified to be the key constituents in the TME by interacting both cancerous cells, healthy cells, tumor-suppressor immune cells, the tumor-promoting immune cells (TAMs, mast cells, Tregs, and pDCs), and by ability of infiltrating throughout the TME. After filtering out the ECM-bound interactions, LGALS9 was found to be the most preferred molecule to construct the interactions. The interactions of LGALS9 showed that TAMs used LGALS9 ligand to communicate with cancer cells, ductal cells, and tumor-suppressor immune cells. Also, our analysis showed that the processes which are linked to ECM remodeling, cell migration, and cellular adhesion might be activated via the interactions of LGALS9. The LGALS9-P4HB interaction which may direct the P4HB-mediated cellular migration was found to be highly active in our analysis. Also, P4HB, as reported to be downregulated gene in PDAC in previous studies, was shown to be upregulated in tumorous samples.

To conclude, we presented the immunosuppressive TME which is derived by the cellular communication in PDAC by using an integrative approach. Each transcriptomics method comes with its own advantages, so integration of data from these methods provided us to interpret the tumor environment in a holistic manner. We explored the immunosuppressive role of TAMs in PDAC and LGALS9 as a key factor in communication of TAMs. To the best of our knowledge, this study is one of the premise works which shows upregulation of P4HB via SC and ST datasets in PDAC. Thus, we suggest that the combinatorial therapies which target LGALS9 and its interaction with P4HB may offer promising outcomes for the treatment of PDAC patients. As an addition, the role of P4HB and LGALS9-P4HB interaction needs to be further investigated in cancer.

## Methods

### Dataset retrieval

The bulk RNAseq data from primary tumor TCGA-PAAD samples were downloaded using TCGAbiolinks R package^80^. The gene expression data of normal pancreatic samples were obtained from the GTEx database^81^. Thus, 178 tumor samples and 332 normal samples (4 from TCGA-PAAD and 328 from GTEx) were acquired. PDAC datasets of scRNA-seq (SC) and ST were downloaded from NCBI database with an accession number of GSE111672 and GSE155698^8,82^. The PDAC-A (scA) and PDAC-B (scB) datasets were obtained from two untreated patients (A and B) with pancreatic ductal adenocarcinoma (PDAC). The tissue samples used for scRNA-seq and ST analysis were taken from the same tumor. scRNA-seq and ST data were processed in parallel^82^. The PDAC-C (scC) which is also downloaded from NCBI database with an accession number of GSE155698 contains scRNA-seq datasets from 14 untreated patients^8^. This dataset has also 3 adjacent normal samples which were also downloaded using the same accession number.

### scRNA-seq preprocess and cell-type annotation

The cell type annotation was performed by using scRNA-seq datasets. Firstly, the genes which were not expressed in any cell were removed. Then, the count values were normalized to the median of total counts of each cell and log transformation was performed. To increase the efficiency of the unsupervised dimension reduction, we detected the highly variable genes in Seurat^83^. In Seurat, a dispersion coefficient is calculated for each gene, and the genes are placed into 20 bins based on their average expression. The dispersion coefficient of each gene is z-normalized, hence the genes with highly variable expression are identified when compared to genes with similar average expression. The method detected 1604, 1567, and 4541 genes as the highly variable in scA, scB, and scC, respectively. Principal component analysis (PCA) was performed on highly variable genes. We than carried out unsupervised clustering with Leiden algorithm, following the non-linear dimensional reduction method UMAP.

The cell type annotation for each cluster were done with the known cell marker genes in scRNA-seq datasets. The cell type marker genes were collected from the previous studies^2,82,84–90^, and databases CellMarker (http://biocc.hrbmu.edu.cn/CellMarker/), Human Protein Atlas (https://www.proteinatlas.org/) and PanglaoDB (https://panglaodb.se). The known signature genes were SLC4A4, FXYD2, SPP1, TFF1, TFF2 and TFF3 (ductal cells); CEACAM5, MSLN, KRT17, LAMC2 and KRT16 (cancer cells); PRSS1, CTRB2 and REG1A (acinar cells); HEPACAM2 and DKK3 (beta cells); HBB and HBA2 (red blood cells, RBCs); TPSAB1 and CPA3 (mast cells); PLVAP and VWF (endothelial cells); IRF7 and GZMB (plasmacytoid dendritic cells, pDCs); ACTA2, FAP, DCN, DKK1 and PDPN (cancer associated fibroblasts, CAFs); CD68 and CD163 (tumor associated macrophages, TAMs); AREG and IL1RL1 (regulatory T cells, Tregs).

### Spatial domain identification

ST datasets were preprocessed by removing the genes with no expression value. Then, the count values were normalized to the median of total counts of each spot before the log transformation. SpaGCN (v.1.2.5) was used to define the spatial domains in each ST datasets. SpaGCN is a graph-based method which integrates gene expression matrix with the spatial location information^91^. The method clusters the spots into the domain by using their gene count profiles and the histological relatedness (spatial location and pixel color intensities). In SpaGCN, the resolution value must be supplied by the user as a hyper-parameter. Optimal resolution parameter for each ST dataset was found by assuming that marker genes should be expressed homogenously within a domain, but differential to other domains. Hence, the marker genes were plotted on the discovered domains at each resolution level, and the resolution level that leads to domains with most homogenous marker gene expression was chosen.

### Analysis of bulk RNA-seq datasets

The bulk datasets (TCGA-PAAD and GTEx pancreas) were retrieved where the gene expression values are given in TPMs. The samples which were labeled as primary solid tumor were selected to be used as the tumor samples, ignoring the other tumor samples. The differentially expressed genes (DEGs) in tumor samples compared to normal samples were identified with the Wilcoxon rank sum test with the p-value adjustment using Benjamini-Hochberg approach. The cut-off *p*-value was 0.01 for the significance of DEGs which resulted with 9822 genes with −1.5<fold change<1.5.

### Functional analysis

One-vs-all DEG analysis was conducted to assign a cell type to each cluster with Wilcoxon rank sum test, and *p*-value correction was carried out by Benjamini-Hochberg approach as done for bulk RNA-seq datasets. The genes with *p*-value smaller than 0.05 was accepted as significant DEGs. The functional analysis was performed to reveal the biological process variations between the clusters by gProfiler. The protein-protein interaction (PPI) network was constructed using Cytoscape 3.9.1 with STRING database^92,93^. The edges were weighted with respect to the STRING confidence scores of the connections.

### L-R pair revealing

One of the most important cellular communication methods is the ligand-receptor (L-R) complexes. The crosstalk via L-R pairs in PDAC samples with scRNA-seq and ST datasets was investigated using CellPhoneDB v3 with its default parameters, permutation analysis was applied for calculating the p-values. The tool retrieves the interacting pairs satisfying the criteria that at least 10% of the cells in the cluster expressed gene of the corresponding ligand or receptor. The interactions with a p-value smaller than 0.05 were selected for further analysis. To combine the information from SC and ST datasets, we detected the top pairs and the top partners of the pairs in both dataset types. The number of interactions between and within the cell types were inspected and the significance of the constructed number of pairs against the whole data were analyzed by permutation test with 1000 iterations. The permutation test was applied to detect if the number of interactions between a pair of cell types was significantly different than the mean of total interactions. A p-value smaller than 0.05 was assumed to indicate the statistical significance. A triangular heat map was generated by annotating if the total number of interactions is larger or smaller than the mean of total interactions.

### Reporting summary

Further information on research design is available in the Nature Research Reporting Summary linked to this article.

### Supplementary information

Supplemental material

Reporting Summary

## Data Availability

The SC and ST datasets which were used in this study can be downloaded from NCBI repository with the accession numbers of GSE111672, GSE155698 and GSE155698.

## Code availability

The codes that were used in this study are available in Github repository https://github.com/avsarg/PDAC_communication.
