# Supplementary material for: An integrated study to decipher immunosuppressive cellular communication in the PDAC environment
Source: NPJ Syst Biol Appl. 2023 Nov 10;9:56. doi: 10.1038/s41540-023-00320-6 (PMC10636193; doi:10.1038/s41540-023-00320-6)
Supplement: Supplementary file 1 — Supplemental material [file 41540_2023_320_MOESM1_ESM.pdf]

Supplementary Figures

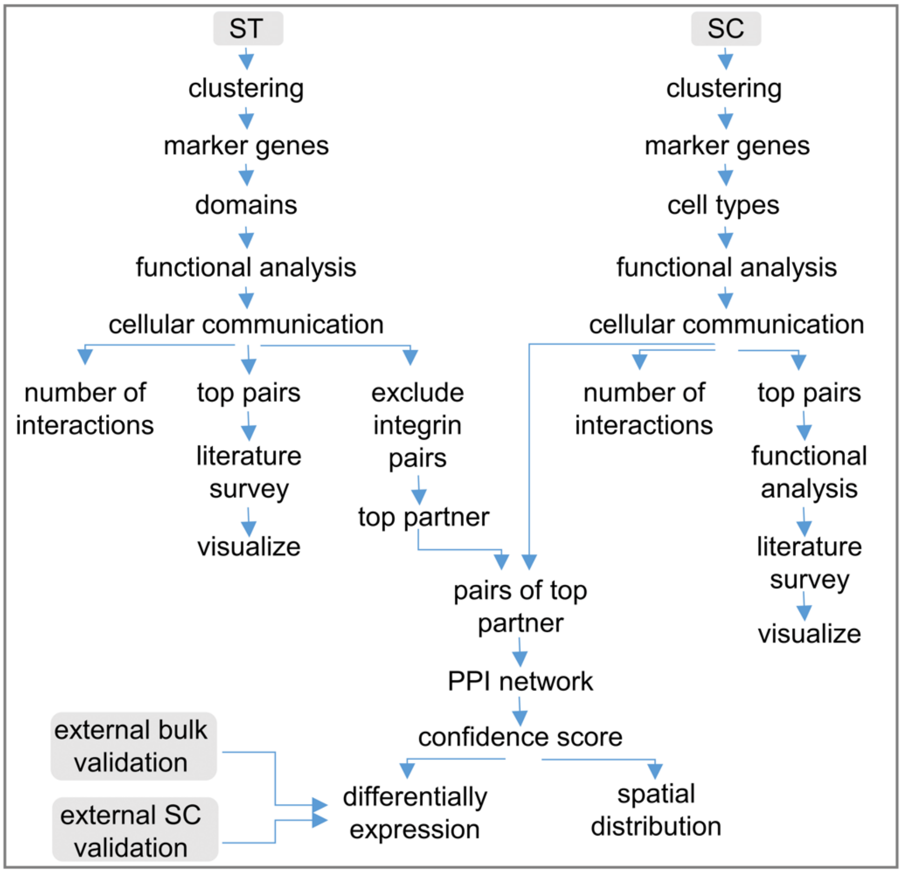

Supplementary Figure 1: The followed approach during the study.

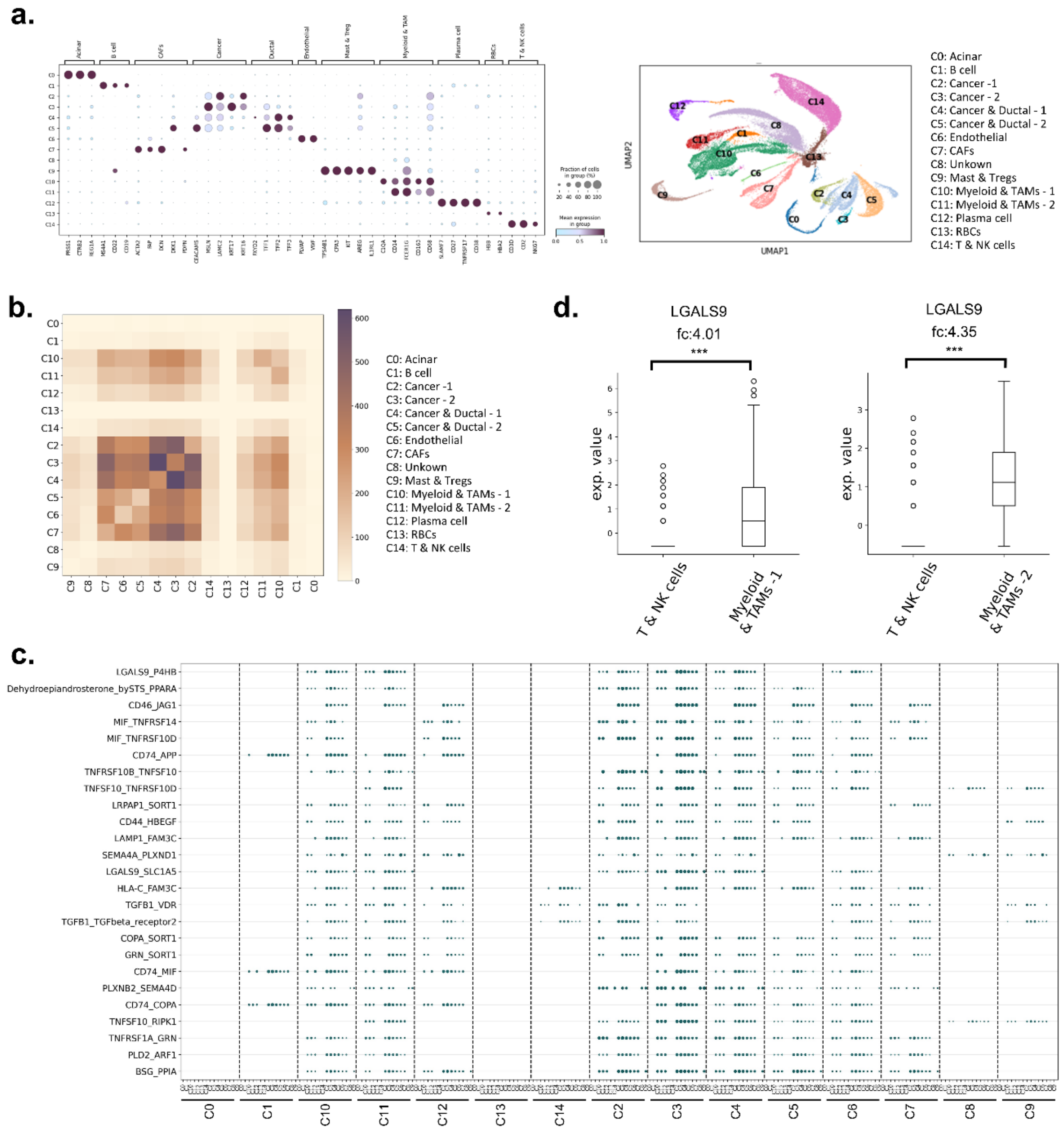

Supplementary Figure 2: Downstream analysis of scC refers that. **a.** the cell clusters and cell type specific marker genes, **b.** the communication dynamics, **c.** the observed top 25 L-R pairs, and **d.** the regulation of LGALS9 compared to tumor-suppressor immune cells and tumor-promoting immune cells.

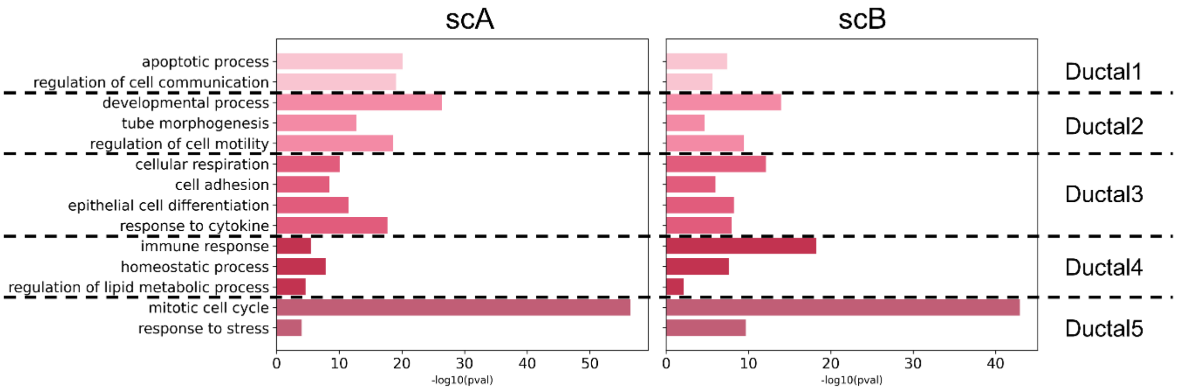

Supplementary Figure 3: GO enrichment analysis of ductal cells.

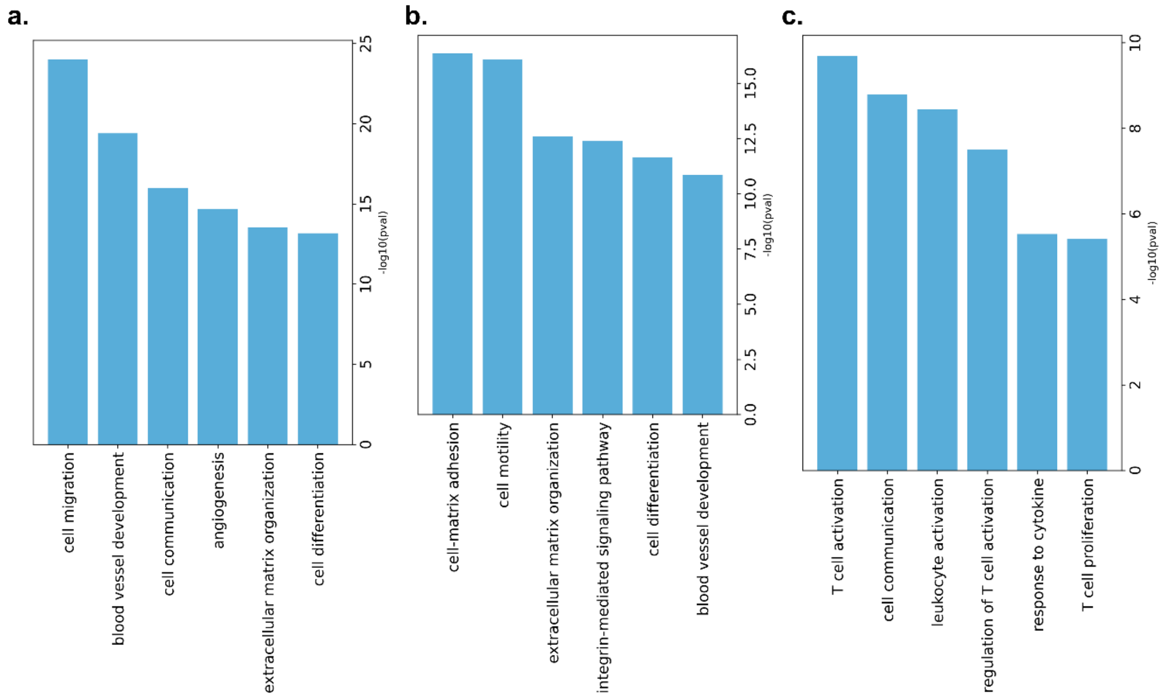

Supplementary Figure 4: GO enrichment analysis for the domains where **a.** cancer specific marker genes high, **b.** fibroblast marker genes are high with cancer marker genes, and **c.** T & NK cells marker genes are high with cancer marker genes.

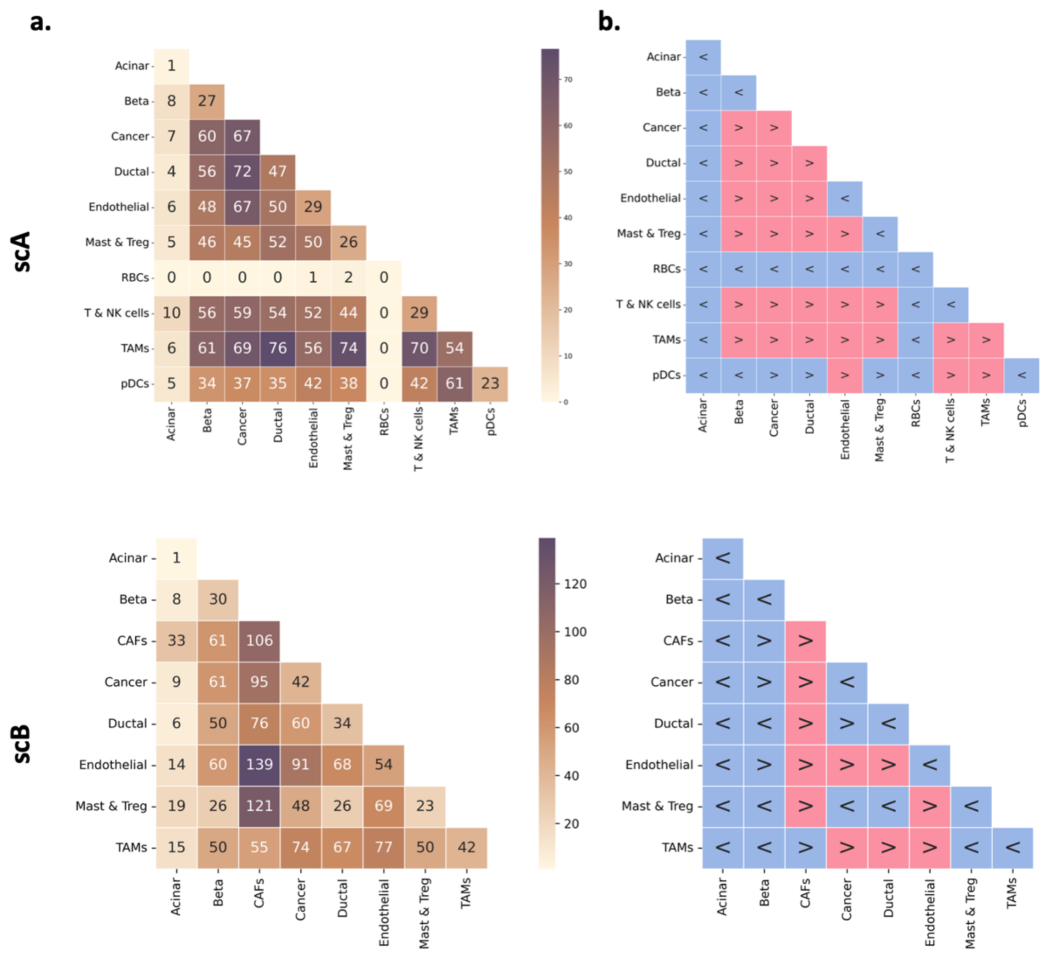

Supplementary Figure 5: Communication profiles in SC datasets with a. total number of interactions which were observed between and within the cell types (the numbers were averaged for ductal cells and cancer cells individually for each sample, and b. the total number of interactions were larger/smaller than the average and significance of their enrichment/depletion (blue refers the p-value > 0.05 while red indicates p-value < 0.05).

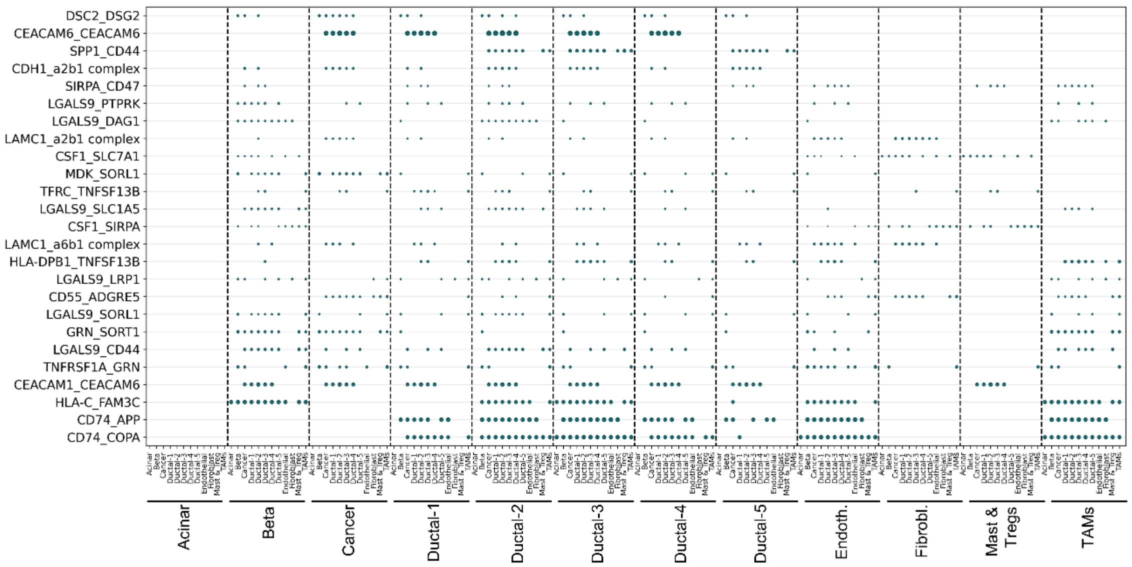

Supplementary Figure 6: The observed top 25 cellular interactions in scB.

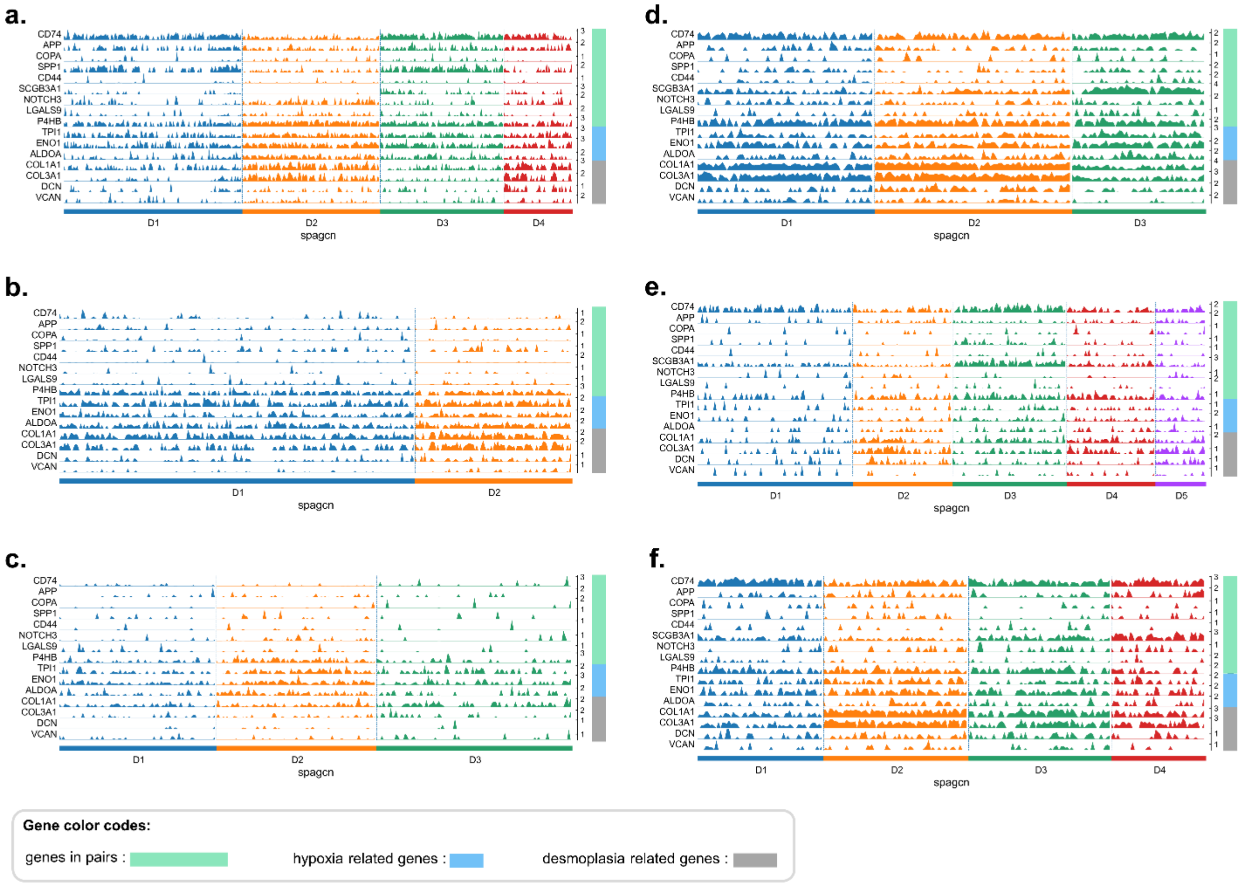

Supplementary Figure 7: Distribution pattern of the genes in mostly observed pairs, hypoxia-related genes, and desmoplasia-related genes in **a.** stA1, **b.** stA2, **c.** stA3, **d.** stB1, **e.** stB2, and **f.** stB3.
